# Assessment of genetic relationships among native and introduced Himalayan balsam (*Impatiens glandulifera*) plants based on genome profiling

**DOI:** 10.1002/ece3.8051

**Published:** 2021-08-26

**Authors:** Helena Korpelainen, Sakina Elshibli

**Affiliations:** ^1^ Department of Agricultural Sciences Viikki Plant Science Centre University of Helsinki Helsinki Finland

**Keywords:** gene flow, genetic structure, genome profiling, genotyping by sequencing, *Impatiens glandulifera*, invasive plants

## Abstract

We conducted genomic characterization based on SNP and SilicoDArT markers on the invasive Himalayan balsam (*Impatiens glandulifera*) plants originating from native and non‐native regions of their distribution. When genetic relationships were explored by PCoA using SNP and SilicoDArT marker data, the first, second, and third principal coordinates explained altogether 37.4% and 31.0% of the variability, respectively. Samples from the UK, Canada, and Pakistan were grouped together, while Indian plants were clearly distinct based on SNP markers but relatively close to the UK–Canada–Pakistan group based on SilicoDArT markers. Constructed trees differentiated individuals into clusters resembling the PCoA patterns. The Bayesian BAPS analysis performed for the SNP data revealed that the individuals were distributed in seven clusters, representing samples from each of the four Finnish populations, India, Pakistan, and the combination of the UK and Canada. Similar clustering was visible in the UPGMA tree. The Indian cluster did not display any ancestral gene flow with the others, while the Pakistani cluster showed ancestral gene flow only with the combined UK and Canada cluster. Furthermore, the latter cluster displayed ancestral gene flow with the Finnish populations varying from 0% to 3.1%. The BAPS analyses conducted for the SilicoDArT data differ slightly: The individuals were distributed in nine clusters, and the Indian cluster exhibited ancestral gene flow with the mixed cluster including Canadian, Pakistani, and UK samples, and one Finnish sample. The AMOVA showed that 45% and 26% of variation was present among the *I*. *glandulifera* groups/populations and the rest within them based on SNP and SilicoDArT markers, respectively. The Bayesian BAPS analyses and the gene flow networks were the most informative tools for resolving relationships among native and introduced plants. It is notable that the small sample sizes for non‐Finnish plant materials may affect the accuracy of the gene flow and other estimates.

## Graphical abstract

We conducted genomic characterization based on SNP and SilicoDArT markers on the invasive Himalayan balsam (*Impatiens glandulifera*) plants originating from the native and non‐native regions of their distribution. Considerable genetic differentiation and the presence of migration between distantly located populations indicate that genetic drift and human activities have facilitated differentiation and further spread of *I. glandulifera*.

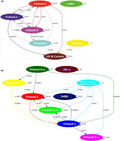

## 1 INTRODUCTION

Nonindigenous species are species distributed outside their historic and native range. Their dispersal may occur either intentionally or accidentally, often being promoted by human activities, such as agriculture, horticulture, aquaculture, transportation, and recreation (Kolar & Lodge, 2001). In certain cases, instead of remaining localized within their new environment, nonindigenous species can become invasive and have large and long‐lasting impacts on the region and its ecosystems, as they increase in number and expand their geographic range. Invasive species also provide interesting opportunities to investigate evolutionary processes (Huey et al., 2005). At introduction, they typically contain just a portion of the species’ gene pool, possibly experiencing a low level of genetic variation and a genetic bottleneck effect. Yet, despite their bottlenecked populations and low evolutionary potential, many invasive species manage to thrive and expand in new environments (Dlugosch & Parker, 2008; Helsen et al., 2019), possibly as a result of the temporal buildup of genetic diversity through multiple introduction events (Helsen et al., 2019) or through phenotypic plasticity facilitating their spread (Skalova et al., 2012).

The Himalayan balsam, *Impatiens glandulifera* Royle (Balsaminaceae), is a tall, self‐pollinated annual plant, which has been introduced widely as a garden ornamental, first to England in 1839 from its native distribution area in Kashmir in the Himalayas (Beerling & Perrins, 1993). Yet, there is some dispute over the date of introduction, as Tanner (2011) has proposed that *I*. *glandulifera* was introduced somewhat prior to 1839, and Morgan (2007) states that the species was first introduced into England from Nepal. Later, due to its popularity as an ornamental plant, high seed production, rapid growth rate, and ability to survive frost, *I*. *glandulifera* has spread throughout Europe and it is invasive in parts of Canada, the United States, and New Zealand, with limited occurrence in Japan (Beerling & Perrins, 1993; Cockel & Tanner, 2012; Perrins et al., 1993; Weber, 2003). It has tendency to form dense monocultures and it is a strong competitor with high plastic responses in regions of introduction (Hulme & Bremner, 2006; Perrins et al., 1993; Skalova et al., 2012). Additionally, Elst et al. (2016) have suggested that differences in habitats between the native and invasive range, especially the higher nutrient availability observed in the new environment, are among main factors driving the invasion of *I*. *glandulifera*. It has become clear that the species represents a significant threat to native ecosystems in many temperate areas of the world (Tanner & Gange, 2020).

Earlier DNA‐based population genetic studies on *I*. *glandulifera* have involved the use of microsatellite markers in investigations conducted in the UK (Provan et al., 2007; Walker et al., 2009). Later, based on microsatellite markers and the sequencing of the nuclear ITS region, Nagy and Korpelainen (2015) compared native and introduced populations of *I*. *glandulifera* and discovered distinct population genetic patterns indicating the strong effect of human‐mediated dispersal. Comparably, Hagenblad et al. (2015) used microsatellites to investigate *I*. *glandulifera* samples from the species’ native range in India and from the introduced range within Europe. They also showed that human activities, at least partially, have facilitated not only introductions, but also further spread of the species across Europe. During recent years, genetic investigations on *I*. *glandulifera* on both native and introduced populations have involved the use of chloroplast DNA, including even the analysis of a complete chloroplast genome from a 125‐year‐old herbarium specimen (Cafa et al., 2020; Kurose et al., 2020). Such studies show that maternal cpDNA provides genetic markers for population studies that could be linked to evolutionary history and phylogeography. Although these markers have been valuable for discovering differentiation and other population genetic processes among *I*. *glandulifera* plants from native and introduced areas, their precision and ability to resolve differences and patterns in genetic structures have not been optimal. During recent years, high‐throughput genotyping‐by‐sequencing (GBS) analyses generating large numbers of markers have become an increasingly frequent approach for molecular characterization and for studies on genetic variation and differentiation (e.g., Ball et al., 2020; Villa‐Machío et al., 2020).

In this study, we employed GBS using both SNP and SilicoDArT marker techniques to perform a comprehensive genome‐wide analysis of the genetic structure on *I*. *glandulifera*. Our aim was to discover two large sets of DNA markers and, by using them, to examine and compare patterns of genetic variability in *I*. *glandulifera* both in the native distribution range and in the area of introduction. We hypothesized that plants in the area of first introduction (England) show a closer genetic relationship to the plants from the native area of distribution (the Himalayas) than those from other regions of introduction, and plants in the areas of introduction show considerable differentiation due to genetic drift and anthropogenic effects, for example, seed dispersal commercially and by home gardeners. Additionally, we hypothesized that the two marker types provide similar results of genetic relationships and differentiation patterns.

## 2 METHODS

### 2.1 Sampling and DNA extraction

The study material of *I*. *glandulifera* representing its native area of distribution included seven samples from India (originating from populations IN‐1, IN‐2, and IN‐3) and five samples from Pakistan (originating from populations PA‐3 and PA‐4), while samples representing introduced material included eight samples from Canada (all from population CA‐3), six samples from the UK (originating from populations UK‐2 and UK‐3), and 68 samples from Finland (originating from populations FI‐1, FI‐2, FI‐3, and FI‐4), a total of 94 samples (Appendix S1, see also Nagy & Korpelainen, 2015). Leaf samples from populations in Finland were placed directly into Eppendorf tubes, let dry in open tubes, and used for DNA extractions within a week, while other samples were squashed onto Whatman FTA paper, let dry in air, and then stored at room temperature for several months before being used for DNA extractions. All DNA extractions were conducted using the E.Z.N.A. Plant DNA Mini Kit Spin Protocol (Omega Bio‐Tek). All DNA concentrations were measured using NanoDrop Spectrophotometer (Thermo Scientific) and adjusted to 20 ng/µl, with a minimum volume of 10 µl. Then, the DNA samples were placed into a fully skirted 96‐well PCR plate, packed, and shipped for genotyping.

### 2.2 Genotyping by sequencing and quality filtering

Genotyping‐by‐sequencing analysis of the 94 *I*. *glandulifera* samples was conducted using a genome profiling service for SNP and SilicoDArT markers by Diversity Arrays Technology Pty Ltd. (Canberra, Australia), following the DArT genotyping protocol of Kilian et al. (2012). In this protocol, DNA samples were exposed to digestion–ligation reactions using restriction enzymes, namely Pst1 in combination with Sphl, with the addition of barcoded adaptors corresponding to the overhangs of the two restriction enzymes. The resulting fragments were amplified by PCR, and the amplicons from each sample were pooled and exposed to cBot (Illumina) bridge PCR and then sequenced using Illumina. The algorithm DArTsoft14 was used to extract both SilicoDArT and SNP markers. Genotyping of ten samples failed due to poor DNA quality (two from India, one from Pakistan, and seven from Canada (Appendix S1). All failed samples were among those stored by squashing onto Whatman FTA paper.

The quality of both SNP and SilicoDArT markers was assessed by different quality parameters, such as call rate and reproducibility percentages. SNP markers were first filtered for all secondary (multiple loci within a fragment that are likely to be linked) and monomorphic loci. Call rate and reproducibility were filtered for both types of markers to the threshold of 0.95. All SNP loci were filtered for deviations from HWE, but no loci were deleted upon filtering. The frequency distribution of polymorphism information content (PIC) values was computed for both marker types after filtering. Filtered data were used for subsequent diversity and genetic structuring analyses. Data filtering was performed using the R 4.0.2 (R Core Team, 2020) package dartR (Gruber et al., 2018).

### 2.3 Genetic diversity and population structure

Genetic diversity indices were calculated using R 4.0.2 (R Core Team, 2020) package dartR (Gruber et al., 2018) for filtered data. An exception was the only remaining Canadian sample, which was excluded from geographic group/population diversity analyses. Diversity indices included the observed (*H*_O_) and expected heterozygosities (*H*_E_), mean within‐population genetic diversity (*H*_S_), total genetic diversity (*H*_T_) and corrected *H*_T_ (*H*_TP_), and the total genetic diversity among populations *D*_ST_ and corrected *D*_ST_ (*D*_STP_) (Nei, 1987). In addition, the fixation index (population differentiation) (*F*_ST_) and corrected *F*_ST_ (*F*_STP_), as well as the inbreeding coefficient (*F*_IS_), were computed (Nei, 1987).

Principal coordinate analyses (PCoA) were applied to investigate genetic relationships among individuals from different groups/populations using R 4.0.2 (R Core Team, 2020) package dartR (Gruber et al., 2018). In addition, Euclidean distance matrices were generated and the corresponding unrooted trees were constructed based on both marker sets using R 4.0.2 (R Core Team, 2020) package dartR (Gruber et al., 2018). A correlation between SNP and SilicoDArT distance matrices was determined by the Mantel test (Mantel, 1967) using the same software.

Population structuring based on both marker types was assessed by the program BAPS 6.0 (Corander et al., 2013; Tang et al., 2009), which uses Bayesian methods to discover population structuring. An admixture analysis based on the mixture clustering of groups of individuals was chosen to estimate the K value that best explains the distribution of the individual samples into different genetic clusters. The analysis was conducted by performing 50 iterations of K (from 2 to 20). The UPGMA trees were constructed based on the Kullback–Leibler divergence matrices that were produced as outputs of the BAPS analyses. Based on the admixture results, the Plot Gene Flow function of the BAPS software was used to estimate and illustrate a network of clusters at the best explaining *K* value.

We used GenAlEx 6.5 (Peakall & Smouse, 2012) to conduct a hierarchical analysis of molecular variance (AMOVA) for both marker types to ascertain the degree of genetic differentiation within and among groups/populations using 999 permutations. The Canadian population was excluded from AMOVA, as it included only one sample after other samples failed in genotyping. Pairwise genetic differentiation among groups/populations was estimated as pairwise *F*_ST_ values.

## 3 RESULTS

**FIGURE 1:**
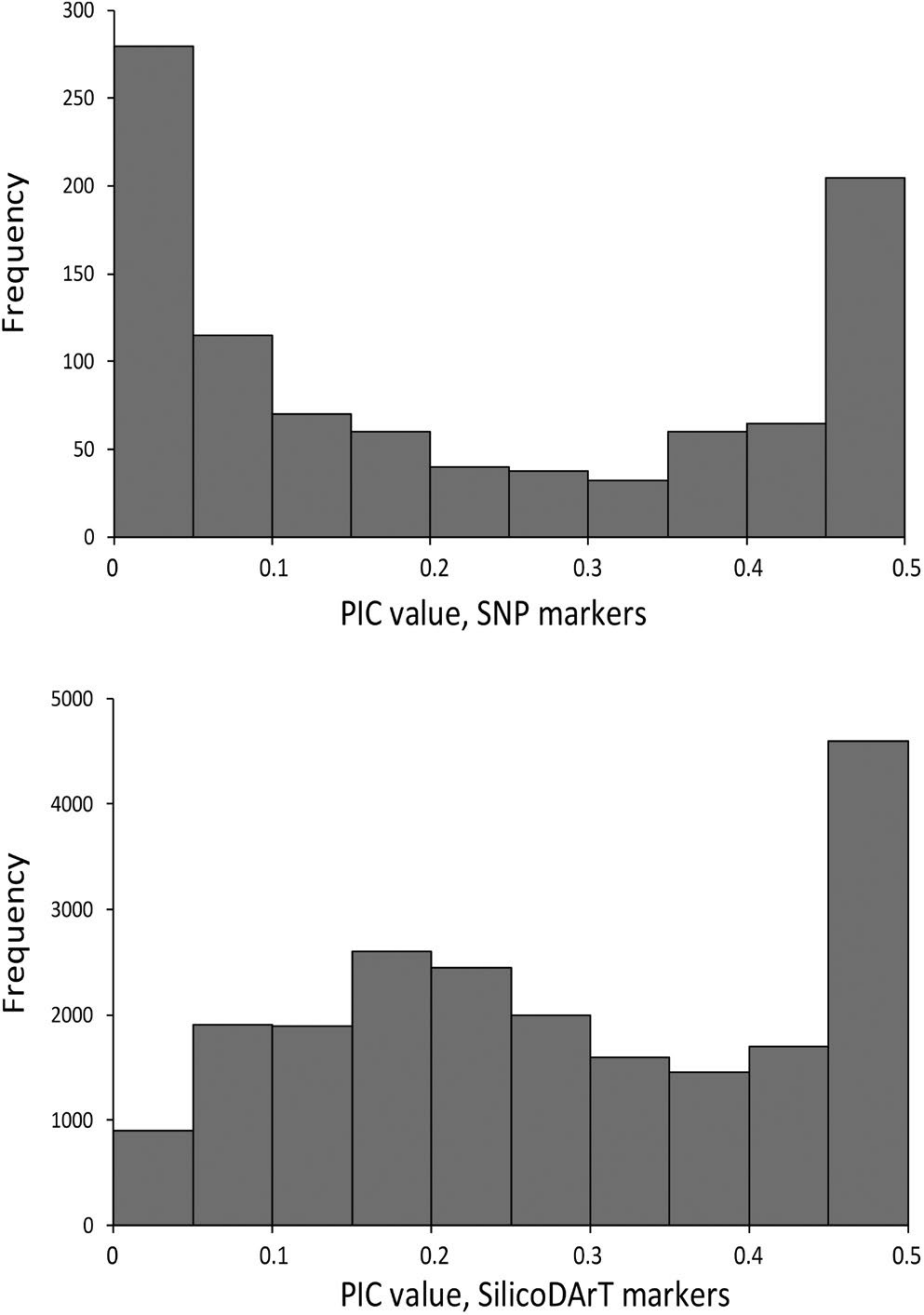
Frequency distribution of PIC values for SNP and SilicoDArT markers

Filtering DArT sequencing data retained 937 SNPs out of original 29,625 markers, and 11,391 SilicoDArT markers out of 21,493 markers for 84 out of 94 individuals sequenced successfully. These data were used to examine patterns of genetic variability in *I*. *glandulifera* both in the native distribution range and in the area of introduction. All markers used in the analyses were filtered for the call rate and reproducibility to the threshold of 0.95. Mean PIC values for SNP and SilicoDArT markers equaled 0.22 and 0.25, respectively. The frequency distribution of PIC values is shown in Figure 1, which shows that values less than 0.05 and greater than 0.45 were most frequent for SNPs, while the frequency distribution was more equal for the PIC values of SilicoDArT markers with values more than 0.45 being the most frequent ones.

The SNP markers showed a higher total genetic diversity and a higher genetic diversity and genetic differentiation among groups/populations compared with SilicoDArt markers, the *H*_T_, *D*_ST,_ and *F*_ST_ values equaling 0.256 (*H*_TP _= 0.273), 0.115 (*D*_STP _= 0.132), and 0.448 (*F*_STP_=0.482) for SNPs, respectively, and 0.166 (*H*_TP _= 0.169), 0.024 (*D*_STP _= 0.028), and 0.146 (*F*_STP _= 0.163) for SilicoDArt markers, respectively. On the other hand, average within group/population diversities was equal, 0.141 and 0.142 for SNP and SilicoDArt markers, respectively. The observed (*H*_O_) and expected heterozygosities (*H*_E_) per population were meaningful only for the four Finnish population samples (FI‐1–FI‐4), for which H_O_ values equaled 0.075, 0.174, 0.132, and 0.074 and *H*_E_ values 0.071, 0.174, 0.132, and 0.111, respectively, based on SNP markers. Thus, only FI‐4 showed a considerably different (lower) value for *H*_O_ in comparison with *H*_E_.

**FIGURE 2:**
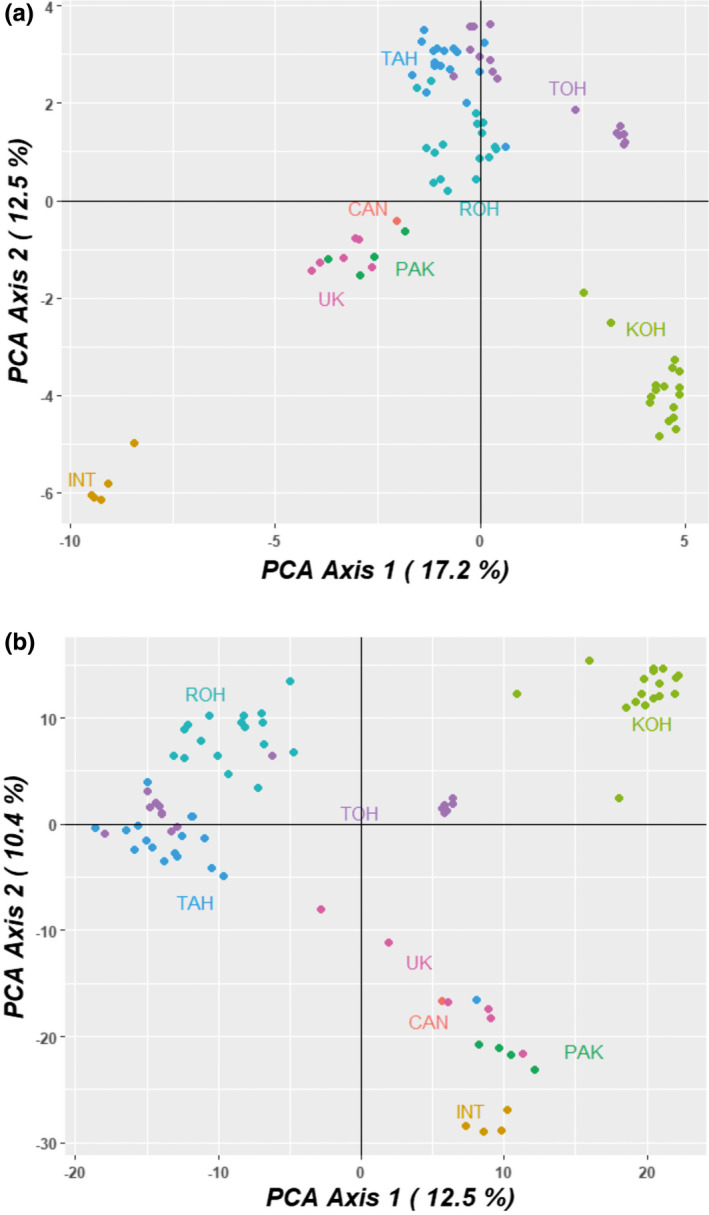
Genetic relationships among 84 *Impatiens glandulifera* as determined by principal coordinates analysis based on SNP (a) and SilicoDArT markers (b). INT = India, PAK = Pakistan, UK = the United Kingdom, CAN = Canada, KOH = Finland‐1, ROH = Finland‐2, TAH = Finland‐3, and TOH = Finland 4

Genetic relationships among individuals within groups/populations were first explored by PCoA. Based on SNP and SilicoDArT marker data, the first, second, and third principal coordinates (the first and second ones are shown in Figure 2) explained 17.2%, 12.5%, and 7.7% and 12.5%, 10.4%, and 8.1%, respectively, altogether 37.4% and 31.0% of the variability. The results showed that ROH, TAH, and TOH samples from Finland grouped together, while individuals from the fourth Finnish population KOH were far apart. In addition, the TOH population possessed two genetic subgroups, and this division was more distinct based on SilicoDArT marker data. Samples from the UK, Canada, and Pakistan grouped together, while Indian plants were clearly distinct based on SNP markers but relatively close to the UK–Canada–Pakistan group based on SilicoDArT markers.

Unrooted trees were constructed based on pairwise genetic distances and cluster analysis, to show the relationships among the 84 genotypes (Appendix S2) and eight groups/population of *I*. *glandulifera* (Appendix S3) based on SNP and SilicoDArT markers. The analysis differentiated the individuals into clusters resembling the patterns observed by PCoA. The four Finnish populations were distinct, but one of them (TOH = FI‐4) clearly showed the presence of two subgroups, similarly as by PCoA. Relationships among individuals from Canada, the UK, India, and Pakistan were not fully consistent and varying slightly depending on the marker type. The only Canadian sample showed a close relationship with most UK samples, and Indian and Pakistani samples were mostly distinct. However, some UK samples showed a closer relationship to the Indian or Pakistani samples than to the rest of the UK samples, differently depending on the marker type, but this may reflect an error in the clustering analysis. Comparably, one of the Finnish samples (from population TAH = FI‐3) showed an inconsistent clustering pattern in SNP‐ and SilicoDArT‐based analyses, showing a close relationship with another Finnish population (TOH) and with UK and Pakistani samples, respectively. Yet, a significant positive correlation was found (*r* = .644, *p* < .001) between SNP and SilicoDArT distance matrices, as determined by a Mantel test, which proved a good fit between SilicoDArT and SNP marker data sets.

**FIGURE 3:**
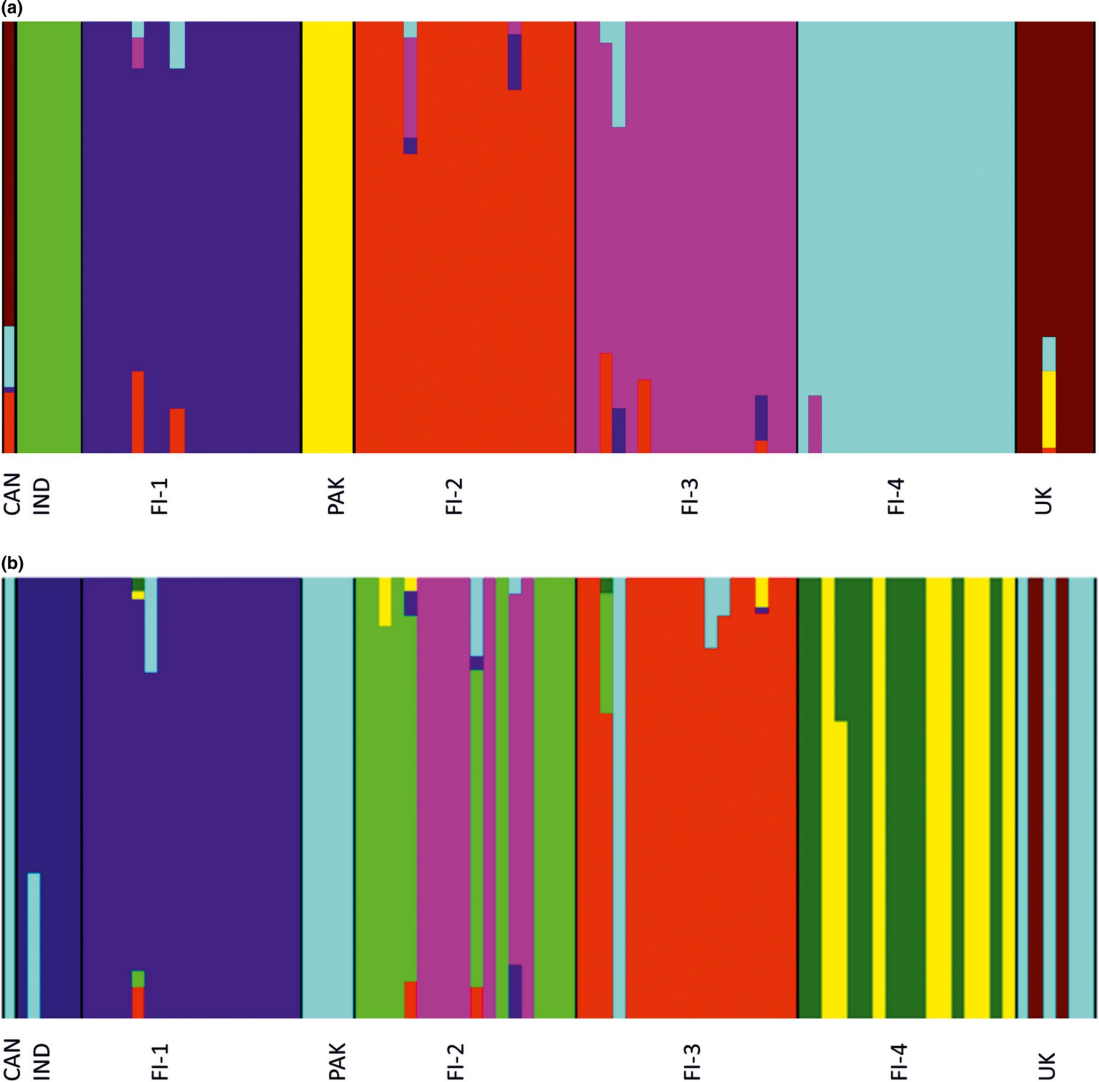
Bar plots showing individual assignment probabilities to genetically distinct clusters of *Impatiens glandulifera* originating from India (IND), Pakistan (PAK), UK, Canada (CAN), and Finland (FI‐1–FI‐4) based on SNP markers (seven clusters; a) and SilicoDArT markers (nine clusters; b)

**FIGURE 4:**
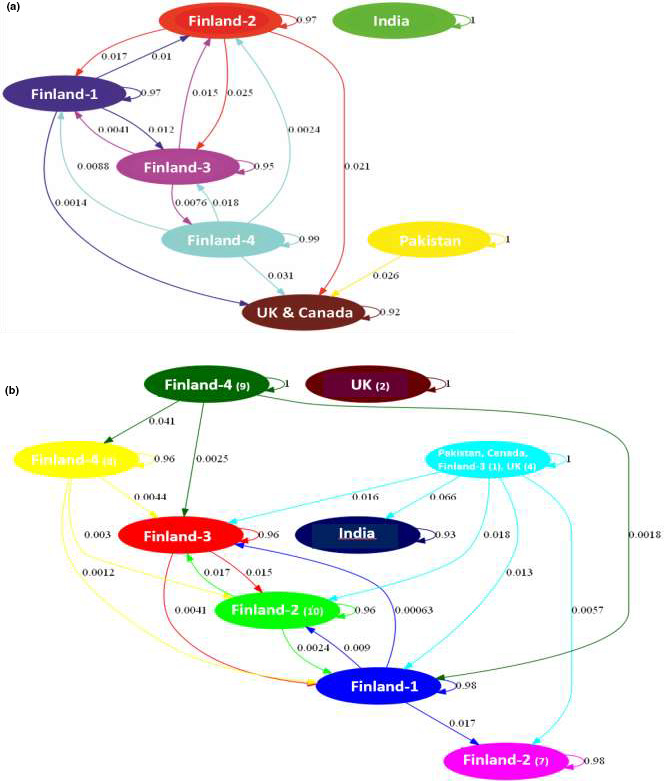
A gene flow network identified for the seven clusters (*K* = 7) of Impatiens glandulifera by BAPS based on SNP markers (a), and for the nine clusters (*K* = 9) based on SilicoDArT markers (b). In parentheses, the numbers of samples in the cluster if the geographic group/population was present in more than one cluster. Gene flow is shown by weighted arrows, which indicate relative average amounts of ancestry coming from the source cluster but present now among individuals assigned to the target cluster

Genetic relationships among the *I*. *glandulifera* genotypes were assessed also using the Bayesian BAPS analysis, which revealed that based on SNP markers the individuals were distributed in seven clusters (Figure 3a), including clusters Finland‐1, Finland‐2, Finland‐3, Finland‐4, UK, and Canada, India, and Pakistan. Similar clustering is also visible in the UPGMA tree constructed based on the divergence matrix, provided as an output of the BAPS analysis (Appendix S4A). The results of the admixture analysis shown as a gene flow network of seven clusters are summarized in Figure 4a. Intercluster ancestral gene flow varied from 0.14% to 3.1%. Among the four populations from Finland, the ancestral gene flow varied between 0.24% and 2.5%. The Indian cluster did not display any ancestral gene flow with the other clusters. The Pakistani cluster showed ancestral gene flow only with the combined UK and Canada cluster (2.6%), which, furthermore, exhibited ancestral gene flow with the Finnish populations varying from 0% to 3.1%. However, the BAPS results based on the SilicoDArT markers differed slightly: The individuals were distributed in nine clusters, as two of the four Finnish populations were split into two clusters and the UK samples were split into two clusters, while the Canadian, Pakistani, and most UK samples, and one Finnish sample from population TAH (FI‐3) formed a mixed cluster (Figure 3b; Appendix S4B). In addition, the Indian cluster exhibited ancestral gene flow (6.6%) with the mixed cluster unlike in the results obtained from SNP‐based analyses, and the cluster composed of two UK samples showed no ancestral gene flow. It is notable that the small sample sizes for non‐Finnish plant materials may affect the accuracy of the gene flow and other group/population‐based estimates.

The AMOVA showed that 45% and 26% of genetic variation lies among the seven groups/populations (*p* < .001; the only Canadian sample excluded) and the rest within them based on SNP and SilicoDArT markers, respectively, which indicates a high degree of differentiation among groups/populations, especially based on SNP marker data (Table 1). Based on pairwise F_ST_ values between groups/populations, all pairs were significantly differentiated from each other (Appendix S5). The differentiation was highest between the Finnish (especially FI‐1) and Indian and Pakistani genotypes, while lowest among three Finnish populations (FI‐2, FI‐3, and FI‐4), based on SNP data, and between plants from the UK, India, and Pakistan, based on SilicoDArT data. Overall, the F_ST_ values did not show very consistent patterns in analyses using SNP and SilicoDArT marker data.

**TABLE 1:** Results of AMOVA conducted for SNP and SilicoDArT marker data, including Indian, Pakistani, UK, and Finnish (four populations) samples. The only Canadian sample was excluded

Origin of variation	*df*	SS	Variance	%	*p*
SNP markers					
Among groups/populations	6	14,571.0	193.2	45	<.001
Within groups/populations	76	17,779.1	233.9	55	<.001
SilicoDArT markers					
Among groups/populations	6	613.9	7.2	26	<.001
Within groups/populations	76	1,559.5	20.5	74	<.001

## 4 DISCUSSION

Our study shows the suitability of SNP and SilicoDArT markers for investigations on *I*. *glandulifera*. Filtering sequencing data retained 937 SNPs out of original 29,625 markers and 11,391 SilicoDArT markers out of 21,493 markers. Such large numbers of markers when compared with previous microsatellite‐, cpDNA‐, or ITS‐based studies (e.g., Cafa et al., 2020; Hagenblad et al., 2015; Kurose et al., 2020; Nagy & Korpelainen, 2015; Provan et al., 2007) provide a good opportunity to obtain precise knowledge of the genetic relationships among plants originating from different geographic regions. As a by‐product of the present genotyping‐by‐sequencing project on *I*. *glandulifera*, we have developed in silico 259 microsatellite markers but those have not been tested otherwise (Korpelainen & Pietiläinen, 2020).

The informativeness of the SNP and SilicoDArT markers was assessed by PIC values, which reveal the diversity detected by these markers. Average PIC values of the SNP and SilicoDArT markers were moderate, 0.22, and 0.25, respectively. Overall, the distribution of PIC values was asymmetrical, values less than 0.05 and greater than 0.45 being most frequent for SNPs, but the frequency distribution being more equal for SilicoDArT markers with values 0.45–0.50 being the most frequent ones. Both lower and higher PIC values of SNP markers compared with SilicoDArT markers have been observed, including, for instance, genomic studies on macadamia (0.21 and 0.29, respectively; Alam et al., 2018), rye (0.37 and 0.22, respectively; Targonska‐Karasek et al., 2017) and on wheat (0.38 and 0.40, respectively; Mahboubi et al., 2020). In *I*. *glandulifera*, despite a lower average PIC value, SNP markers showed a higher total genetic diversity and a higher genetic diversity and genetic differentiation among groups/populations (0.273, 0.132, and 0.482, respectively) compared with SilicoDArt markers (0.169, 0.028, and 0.163, respectively. On the other hand, average within group/population diversities was equal, 0.141 and 0.142, for SNP and SilicoDArt markers, respectively.

Due to the sampling pattern, the observed (*H*_O_) and expected heterozygosities (*H*_E_) per population were meaningful and calculated only for the four Finnish population samples (FI‐1–FI‐4), for which H_O_ values equaled 0.075, 0.174, 0.132, and 0.074, and *H*_E_ values 0.071, 0.174, 0.132, and 0.111, respectively. Thus, only FI‐4 showed a deficiency of heterozygotes compared with the expected heterozygosity. Previously, Nagy and Korpelainen (2015) found that expected heterozygosities were higher in native regions than in regions of introduction. The H_E_ values calculated based on microsatellite data for all native and introduced plants equaled 0.738 and 0.477, respectively. Thus, the marker type effect, that is, the use of highly variable microsatellite markers versus SNPs, was evident. In other microsatellite studies, Hagenblad et al. (2015) have discovered a lower overall variability but, similarly, higher variability in native populations of *I. glandulifera* compared to introduced ones.

When genetic relationships were explored by PCoA based on SNP and SilicoDArT marker data, the first, second, and third principal coordinates explained altogether 37.4% and 31.0% of the variability, respectively. The results showed that three of the four Finnish populations grouped closely together (one showing two clear subgroups), while individuals from the fourth Finnish population were far apart. Samples from the UK, Canada, and Pakistan grouped together, while Indian plants were clearly distinct based on SNP markers but relatively close to the UK–Canada–Pakistan group based on SilicoDArT markers. Overall, the relationship between the native Pakistani samples show a relatively close relationship with the UK and Canadian samples.

Phylogenetic trees constructed to show the relationships among the genotypes differentiated the individuals into clusters resembling the patterns observed by PCoA. The four Finnish populations were distinct from the rest, but one of them showed the presence of two subgroups, similarly as by PCoA. Relationships among individuals were not fully consistent, partly depending on the marker type, despite a significant positive correlation and a good fit found between SNP and SilicoDArT distance matrices. The only Canadian sample showed a close relationship with most UK samples, and Indian and Pakistani samples were mostly distinct. The observed inconsistency may reflect an error in the clustering analysis due to small sample sizes.

Studying the population structure is a key point for understanding patterns of gene flow and for inferring the history of populations. The Bayesian clustering approaches implemented, for example, in BAPS (Corander et al., 2013), are effective and reliable methods for revealing phylogenetic relationships, and population structures. In our study, the BAPS analysis based on SNP data revealed that the individuals were distributed in seven clusters, representing samples from each of the four Finnish populations, India, Pakistan, and the combination of the UK and Canada. Similar clustering was visible in the UPGMA tree constructed based on the divergence matrix, obtained from the BAPS analysis. Intercluster ancestral gene flow varied between 0.14% and 3.1%. The Indian cluster did not display any ancestral gene flow with the other clusters, while the Pakistani cluster showed ancestral gene flow only with the combined UK and Canada cluster. Furthermore, the latter cluster displayed ancestral gene flow with the Finnish populations varying from 0% to 3.1%. However, the BAPS analysis based on the SilicoDArT markers showed the presence of nine clusters, as two of the four Finnish populations were split into two clusters and the UK samples were split into two clusters. The Canadian, Pakistani, and most UK samples, and one Finnish sample from population TAH (FI‐3) formed a mixed cluster. In addition, the Indian cluster exhibited ancestral gene flow (6.6%) with the mixed cluster unlike in the SNP‐based analyses, and the cluster composed of two UK samples showed no ancestral gene flow. Otherwise, intercluster gene flow varied between 0.06% and 4.1%. It is notable that the small sample sizes for non‐Finnish plant materials may affect the accuracy of the gene flow and other group/population‐based estimates. Yet, as revealed by Neophytou (2014) in a simulation‐based investigation, the BAPS software performs well under any sampling scheme, including uneven sample sizes.

The AMOVA showed that 45% and 26% of genetic variation were present among the *I*. *glandulifera* groups/populations and the rest within them based on SNP and SilicoDArT markers, respectively. Thus, the plant groups/populations were highly differentiated, especially when using SNP marker data. Based on pairwise F_ST_ values between groups/populations, all pairs were significantly differentiated from each other. In previous microsatellite‐based studies, lower but still relatively high percentages (24.5% in Nagy & Korpelainen, 2015; 21% and 19% in Helsen et al., 2019) have been found. Differences in marker types and the very high genomic coverage of the present study are reasons for the varied differentiation patterns.

The genome profiling of *I*. *glandulifera* we conducted in the present study showed the relatively close relationship between native Pakistani and introduced UK samples, while the native Indian samples were more distinct, the SNP‐based results showing a clearer pattern compared to SilicoDArT‐based results. The only Canadian sample belonged to the same cluster with the UK samples, while the introduced Finnish populations showed some connections with the UK and Canadian samples, but no clear connection with the native Pakistani and Indian samples, except for one Finnish sample belonging to the mixed Canada, UK, and Pakistan cluster based on SilicoDArT marker data. Thus, the results confirmed our first hypothesis proposing that plants in the area of first introduction (England) show closer genetic relationship to the plants from the native area of distribution in the Himalayas than those from other regions of introduction. The Bayesian BAPS analysis and the following gene flow network were the most informative tools for resolving relationships among native and introduced plants.

Considerable genetic differentiation and the presence of migration between distantly located populations discovered in the present study indicate that genetic drift and human activities have facilitated differentiation and further spread of *I*. *glandulifera* across Europe, thus confirming our second hypothesis. Multiple introductions, considerable genetic differentiation, and drift effects among *I*. *glandulifera* plants across geographic regions have been suggested also in microsatellite‐based studies by Hagenblad et al. (2015), Nagy and Korpelainen (2015) and Helsen et al. (2019) and in a cpDNA sequencing study (Kurose et al., 2020). The results of Helsen et al. (2019) indicate that *I*. *glandulifera* experiences significant gene flow, gradually resulting in higher genetic diversity and lower overall genetic differentiation through time. This combined with phenotypic plasticity (Skalova et al., 2012) may contribute to the successful establishment and further expansion of *I*. *glandulifera* in different regions, consequently increasing its harmful effects on ecosystems.

Additionally, we had hypothesized that the two marker types provide similar results of genetic relationships and differentiation patterns. Overall, this was true. Some detected deviations may at least partly relate to the small sample sizes for non‐Finnish plant materials.

## CONFLICT OF INTEREST

The authors declare no conflict of interest.

## AUTHOR CONTRIBUTIONS

**Helena Korpelainen:** Conceptualization (lead); data curation (lead); formal analysis (equal); funding acquisition (lead); investigation (equal); methodology (equal); project administration (lead); resources (lead); software (supporting); supervision (lead); validation (equal); visualization (supporting); writing—original draft (lead); and writing—review and editing (lead). **Sakina Elshibli:** formal analysis (equal); investigation (equal); methodology (equal); software (lead); validation (equal); visualization (lead); and writing—original draft (supporting).

## Supporting information

Appendix S1

Appendix S2

Appendix S3

Appendix S4

Appendix S5

## Data Availability

Genotyping data are archived in Dryad https://doi.org/10.5061/dryad.dv41ns1xn.
