# Supplementary material for: Assessment of genetic relationships among native and introduced Himalayan balsam (Impatiens glandulifera) plants based on genome profiling
Source: Ecol Evol. 2021 Aug 26;11(19):13295–304. doi: 10.1002/ece3.8051 (PMC8495832; doi:10.1002/ece3.8051)
Supplement: Supplementary file 1 — Appendix S1 [file ECE3-11-13295-s002.docx]

Supporting information Appendix S1.

All *Impatiens glandulifera* samples from India (IN), Pakistan (PA), Canada (CA), Finland (FI) and the UK surveyed using SNP and SilicoDArT markers. In parentheses, the numbers of failed samples.

__________________________________________________________________________________

Population Locality Latitude Longitude Sample size

__________________________________________________________________________________

Native populations

IN-1 Bhrugha Lake N32° 19.142’ E77° 12.357’ 4 (-1)

IN-2 Solang Valley N32° 19.129’ E77° 09.359’ 1 (-1)

IN-3 Chandrkhani Pass N32° 06.482’ E77° 11.532’ 2

PA-3 Naran N34° 53.020’ E73° 38.003’ 2

PA-4 Saiful Malooq National Park N34° 53.780’ E73° 41.489’ 3 (-1)

Introduced populations

CA-3 Nelson, British Columbia N49° 28.290’ W117° 17.338’ 8 (-7)

FI-1 Konala, Helsinki N60° 14.406’ E24° 52.050’ 17

FI-2 Roihuvuori, Helsinki N60° 12.306’ E25° 04.086’ 17

FI-3 Tapanila, Helsinki N60° 16.662’ E24° 59.106’ 17

FI-4 Torpparinmäki, Helsinki N60° 15.366’ E24° 56.256’ 17

UK-2 Camel Trail, Cornwall N50° 20.920’ W04° 47.871’ 5

UK-3 Heathrow, Harmondsworth N51° 29.101’ W00° 28.751’ 1

___________________________________________________________________________________
