# Supplementary material for: Assessment of genetic relationships among native and introduced Himalayan balsam (Impatiens glandulifera) plants based on genome profiling
Source: Ecol Evol. 2021 Aug 26;11(19):13295–304. doi: 10.1002/ece3.8051 (PMC8495832; doi:10.1002/ece3.8051)

Supporting information Appendix S2.

A cluster analysis based on SNP (A) and SilicoDArT markers (B) for grouping 84 *Impatiens glandulifera* genotypes. Two first letters of the sample codes indicate the origin as follows: IN = India, PA = Pakistan, UK = the United Kingdom, CA = Canada, KO = Finland-1, RO = Finland-2, TA = Finland-3 and TO = Finland 4.

A


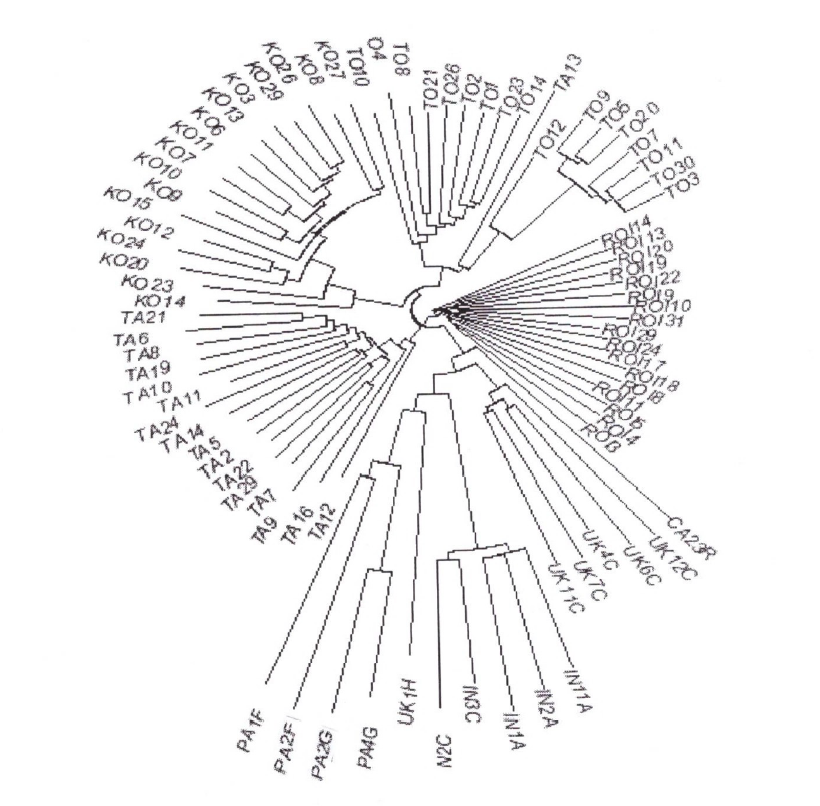


B


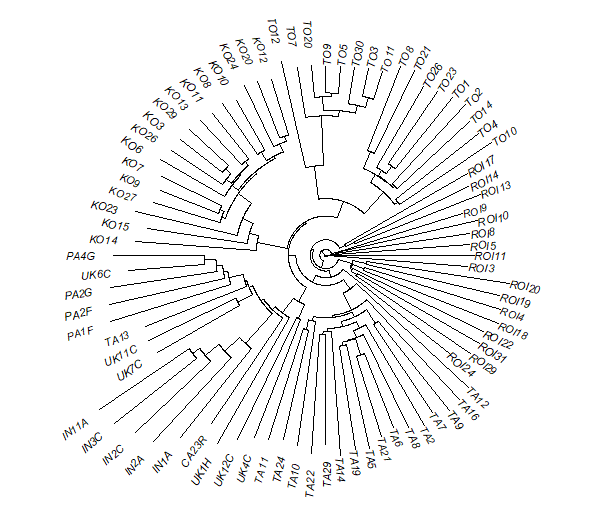

Supplement: Supplementary file 2 — Appendix S2 [file ECE3-11-13295-s003.docx]
