# Supplementary material for: Assessment of genetic relationships among native and introduced Himalayan balsam (Impatiens glandulifera) plants based on genome profiling
Source: Ecol Evol. 2021 Aug 26;11(19):13295–304. doi: 10.1002/ece3.8051 (PMC8495832; doi:10.1002/ece3.8051)

Supporting information Appendix S3.

A cluster analysis based on SNP (A) and SilicoDArT markers (B) for grouping eight *Impatiens glandulifera* groups/population. INT = India, PAK = Pakistan, UK = the United Kingdom, CAN = Canada, KOH = Finland-1, ROH = Finland-2, TAH = Finland-3 and TOH = Finland 4.

A


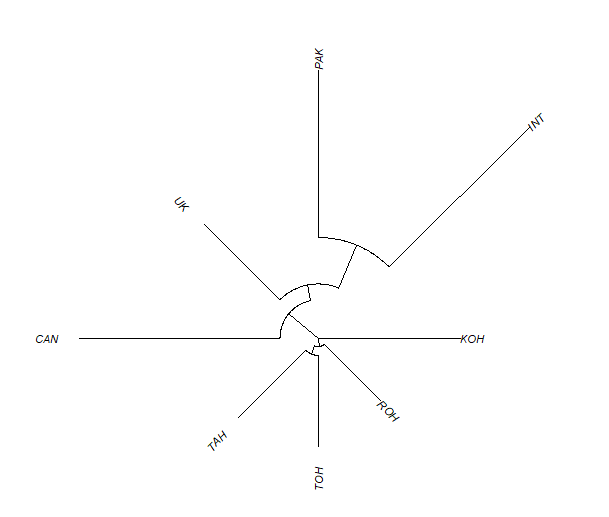


B


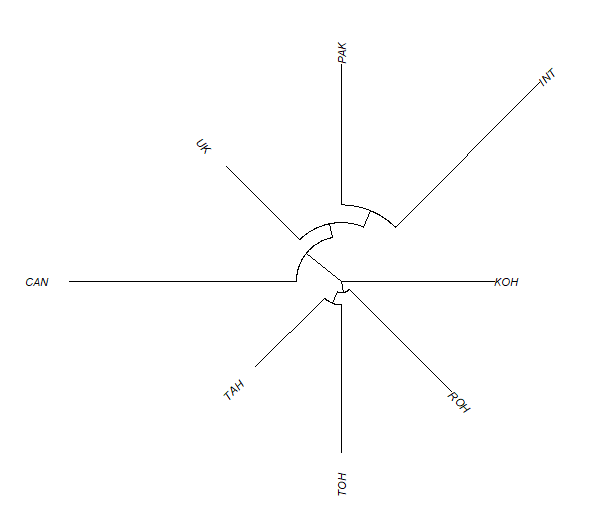

Supplement: Supplementary file 3 — Appendix S3 [file ECE3-11-13295-s001.docx]
