# Supplementary material for: Assessment of genetic relationships among native and introduced Himalayan balsam (Impatiens glandulifera) plants based on genome profiling
Source: Ecol Evol. 2021 Aug 26;11(19):13295–304. doi: 10.1002/ece3.8051 (PMC8495832; doi:10.1002/ece3.8051)

Supporting information Appendix S4.

An UPGMA tree constructed based on the Kullback-Leibler divergence matrix, provided as an output of the BAPS analysis, for *Impatiens glandulifera* groups/populations based on SNP (A) and SilicoDArT markers (B). In parentheses, the numbers of samples in the cluster if the geographic group/population was present in more than one cluster.

A

Cluster 1 = *Finland-2*, Cluster 2 = *India*, Cluster 3 = *Finland-1*, Cluster 4 = *Pakistan*, Cluster 5 = *Finland-3*, Cluster 6 = *Finland-4*, and Cluster 7 = *the UK and Canada*.


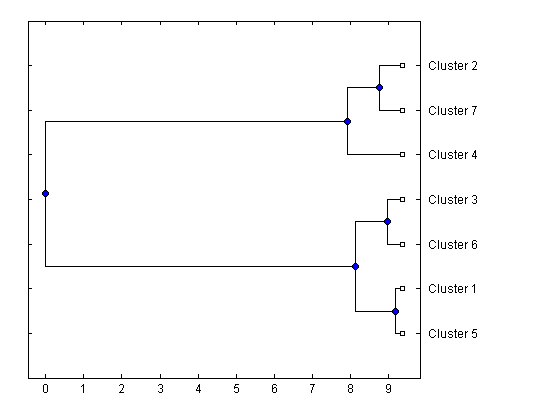


B

Cluster 1 = *Finland-3*, Cluster 2 = *Finland-2* (10 samples), Cluster 3 = *Finland-1*, Cluster 4 = *Finland-4* (8 samples), Cluster 5 = *Finland-2* (7 samples), Cluster 6 = *Canada, Pakistan, the UK* (4 samples) *and Finland-3* (1 sample), Cluster 7 = *the UK (2 samples)*, Cluster 8 = *Finland-4* (9 samples), and Cluster 9 = *India*


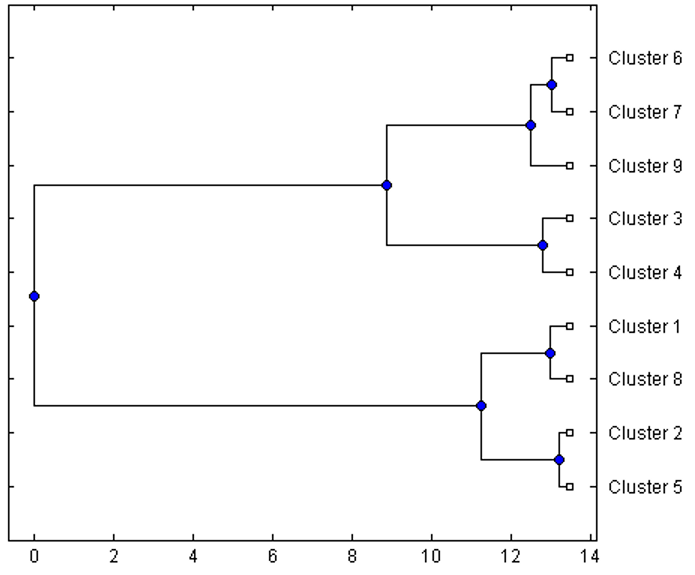

Supplement: Supplementary file 4 — Appendix S4 [file ECE3-11-13295-s005.docx]
