# Supplementary material for: Assessment of genetic relationships among native and introduced Himalayan balsam (Impatiens glandulifera) plants based on genome profiling
Source: Ecol Evol. 2021 Aug 26;11(19):13295–304. doi: 10.1002/ece3.8051 (PMC8495832; doi:10.1002/ece3.8051)
Supplement: Supplementary file 5 — Appendix S5 [file ECE3-11-13295-s004.docx]

Supporting information Appendix S5.

Pairwise F_ST_ values between *I. glandulifera* groups/populations. All values are statistically significant (P<0.05), except for the negative F_ST_ value obtained for the Pakistan-UK pair based on SilicoDArT markers. The only Canadian sample was excluded.

_____________________________________________________________________________

Group/population India Pakistan UK FI-1 FI-2 FI-3

_____________________________________________________________________________

**SNP markers**

India

Pakistan 0.591

UK 0.456 0.394

Finland FI-1 0.749 0.728 0.526

Finland FI-2 0.522 0.471 0.234 0.341

Finland FI-3 0.601 0.568 0.325 0.440 0.184

Finland FI-4 0.652 0.607 0.410 0.415 0.202 0.264

**SilicoDArT markers**

India

Pakistan 0.178

UK 0.182 n/a

Finland FI-1 0.677 0.594 0.481

Finland FI-2 0.380 0.264 0.187 0.384

Finland FI-3 0.450 0.321 0.235 0.471 0.233

Finland FI-4 0.481 0.351 0.267 0.453 0.254 0.266

_____________________________________________________________________________
